# Impact of KRAS Mutations on Management of Colorectal Carcinoma

**DOI:** 10.4061/2011/219309

**Published:** 2011-03-15

**Authors:** Kevin M. Sullivan, Peter S. Kozuch

**Affiliations:** Department of Medicine, Section of Hematology/Oncology, Albert Einstein College of Medicine, Beth Israel Medical Center, Phillips Ambulatory Care Center, 10 Union Square East, Suite 4C, NY 10003, USA

## Abstract

The epidermal growth factor receptor (EGFR) pathway is a therapeutic target in the management of colorectal cancer (CRC). EGFR antagonists are active in this disease; however, only a subset of patients respond to such therapy. A Kirsten ras sarcoma viral oncogene (KRAS) wild-type (WT) status of the tumor is necessary, but possibly not sufficient, for a response to anti-EGFR monoclonal antibody therapy. Mechanisms of primary resistance to such therapy in patients harboring KRAS WT tumors are discussed. Strategies to overcome resistance to anti-EGFR monoclonal antibody therapy, including novel agents and combinations of novel therapies, are explored. Also, the use of anti-EGFR monoclonal antibodies in the adjuvant and neoadjuvant setting is reviewed.

## 1. Introduction

**Figure 1:**
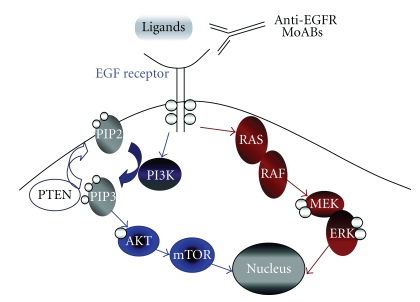
EGFR signaling pathway [4]. (Reprinted with permission from American Society of Clinical Oncology 2008. All rights reserved.)

Tumor growth and progression depends in part on the activity of cell surface membrane receptors which control signal transduction pathways. These growth factor receptors can have aberrations in their expression and regulation and activation of growth factor pathways is common in many malignancies [1]. The EGFR, a transmembrane glycoprotein also called ERBB-1 or HER1, is a member of a family of receptor tyrosine kinases (TKs). The EGFR is involved in signaling pathways controlling cell growth, differentiation, and proliferation and is expressed in many different types of normal tissues as well as several tumor types, including CRC [2, 3]. Figure 1 illustrates the main EGFR signaling pathways described [4]. When a ligand binds to the EGFR, the receptor forms a dimer resulting in a signaling cascade within the cell via tyrosine kinase activity [5]. This signaling cascade occurs by the activation of receptor autophosphorylation which triggers a number of intracellular pathways regulating cell proliferation, prevention of apoptosis, and promotion of invasion, metastasis, and neovascularization [6]. The proto-oncogene c-erb-B encodes the EGFR, and activation of the proto-oncogene results in EGFR expression in many tumors [7, 8]. There was therefore interest in investigating this pathway as a potential anticancer therapy target. 

Pharmacologically, there are two classes of EGFR antagonists currently in clinical use: antiEGFR monoclonal antibodies directed against the extracellular domain of the receptor and oral small-molecule EGFR TK inhibitors which block the receptor TK activity competitively [10]. The antiEGFR monoclonal antibodies, cetuximab and panitumumab, act by binding to the extracellular region of the EGFR and therefore block the ligand-binding region which prevents ligand-induced TK activation [11]. These monoclonal antibodies solely recognize the EGFR, making them very selective for their target [5]. The small-molecule EGFR TK inhibitors, erlotinib and gefitinib, inhibit the catalytic activity of the TK by competing with adenosine triphosphate (ATP) to bind to the intracellular domain [10]. These small-molecule inhibitors are not exclusive to the EGFR pathway and can block different receptor tyrosine kinases, such as the vascular endothelial growth factor (VEGF) receptor and other members of the EGFR family. 

**Figure 2:**
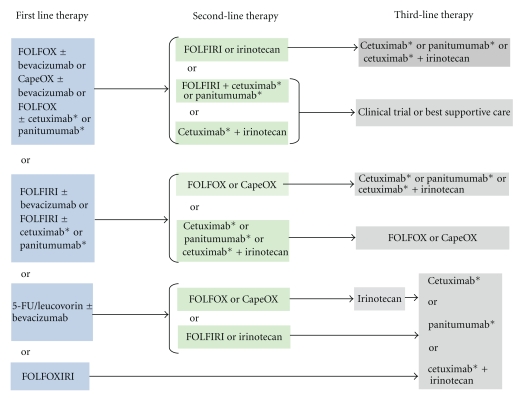
The current treatment paradigm for patients with metastatic colorectal cancer who are appropriate for intensive therapy [9]. *For patients with KRAS WT gene only. CapeOX: capecitabine + oxaliplatin.

Anti-EGFR monoclonal antibodies have been evaluated in both untreated metastatic CRC and chemotherapy refractory disease. Figure 2 summarizes the current treatment paradigm for metastatic colorectal cancer including the appropriate incorporation of antiEGFR monoclonal antibody therapy which improves survival for appropriately selected patients [9]. Table 1 summarizes selected clinical trials of antiEGFR monoclonal antibodies in metastatic CRC. Response rates with single-agent antiEGFR monoclonal antibodies range from 9–12%, with much higher response rates observed when cetuximab is used in combination with chemotherapy [12–22]. When administered to unselected metastatic CRC patients, only a minority responded to EGFR inhibitor therapy. Therefore, a method to identify and predict sensitivity to these drugs was needed. 

## 2. Prediction of Response to Anti-EGFR Monoclonal Antibodies

The RAS family of proto-oncogenes include HRAS, KRAS, and NRAS [23]. KRAS (Kirsten ras sarcoma viral oncogene) is a guanosine triphosphate-(GTP-) binding protein downstream of the EGFR and is a central component of the mitogen-activated protein kinase (MAPK) pathway, a component of the EGFR signaling cascade [23]. Roughly 40% of colorectal cancers are characterized by a mutation in the KRAS gene [24]. About 90% of these mutations occur in codons 12 and 13 in exon 2 of the KRAS gene, with the remaining mutations occurring in codons 61 and 146 (roughly 5% each) [25, 26]. Such KRAS mutations lead to EGFR-independent constitutive activation of the signaling pathway and predict for a lack of response and benefit from antiEGFR monoclonal antibodies cetuximab and panitumumab [27–35]. De Roock et al. showed that codon 61 mutations predicted for lack of response to cetuximab similar to codon 12 and 13 mutations; however, codon 146 mutations did not affect cetuximab efficacy [26]. Failure to test for codon 61 mutations may miss a significant mutation which would confer resistance to antiEGFR monoclonal antibody therapy. There is a very high concordance of KRAS mutational status between the primary tumor and metastasis, ranging from 92–100% [36–38]. However, KRAS mutation status heterogeneity between primary tumors, lymph nodes and distant metastases in 5–10% of patients has been reported, with mixed responses to antiEGFR monoclonal antibody therapy in those with metastatic CRC [37, 39, 40]. Because of this, some clinicians have called for a reassessment of KRAS mutation status on metastatic foci in situations where only the primary tumor was assessed for KRAS status [41]. 

Table 2 summarizes clinical trials of antiEGFR monoclonal antibodies which included analysis of treatment effect and KRAS mutation status. Amado et al. assessed the predictive role of KRAS mutational status in a randomized phase III trial comparing panitumumab monotherapy with best supportive care (BSC) in patients with chemotherapy refractory metastatic CRC [24]. This trial showed that the clinical benefit associated with panitumumab was restricted to the KRAS WT population. KRAS mutations predicted for lack of clinical benefit to panitumumab [24]. Similarly, Karapetis et al. showed that treatment with cetuximab significantly improved OS and PFS in patients with KRAS WT tumors; however, in this chemotherapy-resistant patient population, those with mutated KRAS tumors did not benefit [38]. Use of cetuximab as first-line treatment for metastatic disease was investigated by Van Cutsem et al.; patients were randomly assigned to receive FOLFIRI with or without cetuximab [36]. A statistically significant benefit in PFS for patients with KRAS WT tumors receiving cetuximab and chemotherapy was confirmed in a final presentation of this trial [42]. Bokemeyer et al. investigated the use of cetuximab in combination with FOLFOX chemotherapy as initial treatment for metastatic disease [34]. A retrospective analysis of this data revealed that cetuximab and chemotherapy had a statistically significant increased response rate and lower risk of disease progression compared with chemotherapy alone in patients with KRAS WT tumors [43]. Prospectively, panitumumab has been investigated with either FOLFOX or FOLFIRI chemotherapy in the first-line metastatic setting [44, 45]. The addition of panitumumab to FOLFOX chemotherapy was associated with a statistically significant improvement in PFS [44]. Taken together, the data in Table 2 supports that in metastatic CRC, KRAS WT and mutation status predict for potential sensitivity to, and definite resistance to, respectively, both antiEGFR monoclonal antibodies, regardless of prior treatment and irrespective of use as monotherapy or in combination. Notably, while KRAS status is an established predictor of response to antiEGFR monoclonal antibody therapy, it has been disproven as a prognostic marker. In contrast to KRAS mutational status, evaluation of EGFR expression of CRC cells has failed to demonstrate predictive value for antiEGFR monoclonal antibody therapy. Cunningham et al. reported that the intensity of EGFR staining by immunohistochemical analysis did not correlate with response rate to cetuximab [13]. Similar data has also been reported with panitumumab [46]. KRAS mutated CRC absent of antiEGFR monoclonal antibody therapy is not inferior to patients with KRAS WT disease. The evaluation of KRAS mutational status is a mandatory aspect of management of patients at the time of diagnosis of metastatic CRC. 

## 3. Mechanisms of Resistance

While KRAS mutations are a major mechanism of primary resistance to antiEGFR monoclonal antibody therapies, resistance mechanisms in KRAS WT patients are also being defined. Forty-sixty percent of patients with KRAS WT tumors fail to respond to treatment with antiEGFR monoclonal antibodies [51]. Therefore, other possible molecular determinants of response are being identified in those patients with EGFR monoclonal antibody-resistant WT KRAS disease. 

The importance and frequency of NRAS (a ras oncogene variant) mutations in CRC remains under-investigated [52, 53]. Lambrechts et al. found that NRAS, KRAS, and BRAF mutations were all mutually exclusive events, with combined WT status of these genes associated with higher response rates and longer progression-free survival times [54]. Lambrechts et al. also reported that an NRAS mutation was associated with a lack of response to cetuximab. Irahara et al. investigated the relationship between NRAS mutations and clinical outcome in a collection of 225 colorectal cancers from two prospective cohort studies [55]. NRAS mutations were detected in 2.2% of the CRCs. There was no apparent association between the NRAS mutations and any clinical or pathologic features, including patient survival. However, the low frequency of NRAS mutations may obscure a significant relation. De Roock et al. conducted a retrospective analysis of over 700 tumor samples from patients treated with cetuximab plus chemotherapy and found a NRAS mutation frequency of 2.6%. Additionally, in KRAS wild types, carriers of NRAS mutations had a significantly lower response rate to cetuximab than NRAS wild types (7.7% versus 38.1%, *P* = .013) [26]. There was, however, no significant difference in median PFS (14 versus 26 weeks, *P* = .055) and median OS (38 versus 50 weeks, *P* = .051) between NRAS wild types and mutants [26]. 

B-type Raf kinase (BRAF) is a component of the RAS-RAF-MEK signaling cascade of the EGFR (see Figure 1) [56]. A specific mutation in the BRAF gene (V600E) is present in approximately 5–8% of CRCs and is thought to be limited to those tumors without mutations in exon 2 of KRAS [42, 57]. BRAF, which is located directly downstream from RAS, can have activating mutations leading to stimulation of the MEK pathway [56, 58]. BRAF mutations appear to confer a poor prognosis, and it appears that BRAF mutations also predict for a lack of response to antiEGFR monoclonal antibodies [42, 57, 59, 60]. Loupakis et al. analyzed 87 patients with KRAS WT tumors for the BRAF V600E mutation who were receiving irinotecan and cetuximab for refractory metastatic CRC. This mutation was found in 15% of the patients and was associated with a lack of response to therapy (0% versus 32%, *P* = .016) and a shorter overall survival (4.1 months versus 13.9 months, *P* = .037) [61]. An additional retrospective analysis of 113 patients treated with antiEGFR monoclonal antibodies found the V600E BRAF mutation in 14% of the KRAS WT patients and was associated with no response to therapy and a statistically significant shorter progression-free survival and overall survival compared with BRAF WT patients [59]. In De Roock's retrospective analysis of tumor samples from patients treated with cetuximab plus chemotherapy, a BRAF mutation was discovered in 4.7% of tumors [26]. In KRAS wild types, carriers of BRAF mutations had a significantly lower response rate to cetuximab than in BRAF wild types (8.3% versus 38.0%, *P* = .0012), a significantly shorter PFS (8 versus 26 weeks, *P* < .0001), and a significantly shorter OS (26 versus 54 weeks, *P* < .0001) [26]. KRAS and BRAF mutation status do not, however, appear to affect the clinical benefit of oxaliplatin or irinotecan on PFS or OS [62]. Several compounds (PLX4032, PLX4720, and GDC-0879) which selectively inhibit the kinase enzyme BRAF containing the V600E mutation are in clinical development [63]. In BRAF mutant cancer cell lines, these selective BRAF inhibitors potently block RAF-MEK-ERK signaling. However, in those tumors that are BRAF WT, but possess a KRAS mutation, these BRAF inhibitors activate this same pathway and therefore should be avoided in those cancers with RAS mutations [64–66].

The mitogen-activated protein kinase kinase (MEK, also known as MAP2K) is downstream of BRAF in the RAS-RAF-MEK signaling cascade of the EGFR and uses extracellular signal-regulated kinase (ERK) as a substrate (see Figure 1) [67]. A number of MEK inhibitors such as AS703026, AZD6244 and RO5068760 have been or currently are being investigated in phase 1 and 2 clinical trials [68, 69]. The development of several MEK inhibitors has been halted because of either very low response rates or due to ocular toxicity [70]. These agents have however shown substantial preclinical activity in tumor cell lines harboring the BRAF V600E gene mutation [71]. It has been established that KRAS has a number of downstream effectors that are not blocked by MEK inhibition, and indeed BRAF mutant cell lines were found to be more sensitive to MEK inhibitors than KRAS mutant cells [71]. It is imperative to be able to identify which patients are likely to respond to MEK inhibitors, and it appears that those with BRAF mutations are a good start. Given that KRAS signaling operates through a number of downstream effectors, those with KRAS mutations may require a combination of targeted agents. Preclinical evidence suggests that BRAF gene amplification is a mechanism of resistance to both MEK and BRAF inhibitors and a combination of these inhibitors may be a strategy to overcome this [72]. 

An additional EGFR pathway is the PTEN/PI3K/AKT pathway [phosphatase and tensin homologue gene (PTEN)]. PTEN encodes a phosphatase which uses phosphatidylinositol-3,4,5-triphosphate (PIP-3) as a major substrate [73]. Loss of PTEN function leads to increased PIP-3 concentration, with resultant AKT hyperphosphorylation protecting tumor cells from apoptosis [73]. Roughly 60% of primary CRCs have a hyperphosphorylated AKT [74]. PTEN loss, activating mutations of phosphatidylinositol 3-kinase catalytic alpha polypeptide (PIK3CA) and activating mutations in KRAS/BRAF/MAPK confer resistance to apoptosis induced by cetuximab [75]. In patients with KRAS WT tumors treated with a cetuximab-based regimen, PTEN loss was associated with a significantly shorter OS [60]. Approximately one third of CRCs harbor activating somatic mutations in PIK3CA, and it has been reported that these mutations are predictive of lack of benefit from antiEGFR therapy [76]. Additional genetic alterations which could confer resistance to antiEGFR monoclonal antibodies include an inhibitor of PI3K signaling; coamplification of PAK4 (p-21-activated protein kinase) and AKT, which are downstream mediators of PI3K signaling; and amplification of IRS2 (insulin receptor substrate 2), which is an upstream activator of PI3K signaling [77, 78]. 

## 4. Strategies to Overcome Resistance

A number of approaches to the problem of resistance to antiEGFR monoclonal antibody therapy have been studied and are ongoing. Combining antiEGFR monoclonal antibodies with cytotoxic chemotherapy has already been discussed. Erlotinib and gefitinib, two oral small molecule EGFR inhibitors, are inactive by themselves [79, 80]. The combination of erlotinib with capecitabine and oxaliplatin in previously treated patients and the combination of gefitinib with FOLFOX were investigated in small phase II studies with favorable results, however randomized trials with chemotherapy alone as a control are needed [81–83]. Dual antiEGFR therapy with antiEGFR monoclonal antibodies plus antiEGFR TK inhibitors may overcome resistance to either drug alone. A 41% response rate was reported for the combination of cetuximab and erlotinib in patients with refractory disease, however this was limited to patients with KRAS and BRAF WT tumors [84]. 

EGFR and vascular endothelial growth factor (VEGF) have several signal transduction pathways in common, with preclinical data revealing that antiEGFR and antiVEGF drug combinations have synergistic activity [85]. The BOND-2 study randomized patients with irinotecan- and oxaliplatin-refractory but bevacizumab naïve disease to cetuximab and bevacizumab with or without irinotecan [21]. Response rates, TTP and OS favored the triple drug regimen, however, these results did not hold up in a subsequent study of this combination [21, 22]. Two subsequent randomized phase III trials have shown that combinations of antiEGFR monoclonal antibodies plus bevacizumab do not improve outcomes and can actually cause increased toxicity irrespective of KRAS mutational status. The PACCE trial evaluated panitumumab combined with oxaliplatin- or irinotecan-based chemotherapy plus bevacizumab. The dual monoclonal antibody arm was associated with increased toxicity and significantly shorter PFS in patients with both KRAS WT and mutant tumors [50]. Similar results were observed with the combination of cetuximab to a regimen containing capecitabine, oxaliplatin, and bevacizumab in the CAIRO2 trial [49]. 

Novel agents and combinations are being employed in an attempt to overcome antiEGFR monoclonal antibody resistance. Motesanib, an oral inhibitor of VEGF, platelet derived growth factor (PDFG) and Kit receptors is being investigated with or without panitumumab in patients with refractory disease [86]. A number of inhibitors of the mutant BRAF kinase are in clinical development, as discussed above [87]. AMG 102 is an investigational monoclonal antibody against human hepatocyte growth factor (also known as cMET, of which overexpression correlates with cetuximab resistance) is being studied in combination with panitumumab in patients with metastatic CRC [88, 89]. 

## 5. Neoadjuvant and Adjuvant Therapy

Given the clinical benefit of antiEGFR monoclonal antibodies in patients with metastatic disease, evaluation of these therapies as postoperative (adjuvant) treatment was warranted. In the adjuvant setting, eradication of micrometastatic disease is associated with increased cure rates. N0147 randomized 1760 patients with resected stage III KRAS WT colon cancer to FOLFOX with or without cetuximab [90]. Interim analysis led to premature closure of this trial after it was determined that no group of patients benefited from cetuximab [90]. Initially this trial enrolled patients regardless of KRAS mutational status, and among 658 patients with mutant KRAS, the addition of cetuximab to FOLFOX resulted in impaired disease-free survival (DFS) and a trend toward impaired OS [91]. 

 In patients with rectal cancer, EGFR is a logical target in combination with neoadjuvant radiotherapy (RT). Retrospective analyses have demonstrated lower pathologic complete response (pCR) rates and shorter DFS in patients with rectal cancer expressing EGFR who were treated with neoadjuvant RT, suggesting that radiosensitivity might be increased by targeting the EGFR [92, 93]. Several phase I/II studies have investigated the combinations of cetuximab and chemoradiotherapy in the neoadjuvant setting for patients with rectal cancer. These studies have demonstrated that cetuximab could be safely combined with preoperative chemoradiotherapy but the pCR rates have been low (5–12%) [94–100]. In two of these studies [96, 99], subsequent analyses were done to correlate KRAS mutation status with response rate. Among patients with KRAS WT tumors, Bengala et al. reported a trend toward a greater rate of tumor regression (36.7% for KRAS WT versus 11% for KRAS mutant), however it did not reach statistical significance (*P* = .12) [101]. Debucquoy et al. also did not find a correlation between KRAS WT tumors and pathologic response to therapy [102]. To our knowledge, panitumumab has not been studied in combination with RT in patients with rectal cancer. Given the failure of antiEGFR monoclonal antibodies to demonstrate a benefit in the adjuvant setting for stage III WT KRAS colon cancer, the value of further study of these agents for rectal cancer is doubtful. 

Preclinically gefitinib has demonstrated improved radiosensitization [103]. Valentini et al. investigated the combination of gefitinib, continuous infusion 5-fluorouracil (5-FU) and pelvic RT in 41 patients with locally advanced rectal cancer and reported a pCR rate of 30%, however toxicity was an issue and further studies are necessary to establish the safety of this combination [104]. 

The effect of combined antiEGFR and antiVEGF therapy in combination with preoperative chemoradiotherapy for rectal cancer remains unknown, however given the negative results reported for combined EGFR and VEGF blockade in patients with metastatic CRC in combination with chemotherapy, studies investigating this avenue are unlikely [22, 49, 50]. Blaszkowsky et al. performed a small study investigating the combination of bevacizumab, erlotinib and 5-FU with RT in patients with locally advanced rectal cancer [105]. The regimen was found to be well-tolerated and highly active with a pCR rate of 47% and may deserve further investigation. However, the value of pCR as a surrogate for DFS and OS is uncertain. 

## 6. Conclusion

Anti-EGFR monoclonal antibodies are among the standard treatment options for patients with metastatic CRC given their established efficacy. It is now clear that the benefit of antiEGFR monoclonal antibodies is isolated to patients with KRAS WT tumors. It appears that KRAS mutational status is just the beginning of our understanding of the EGFR as an integral component of the biology of CRC. Given that only a subset of patients respond to antiEGFR therapy, there is a need for better predictors to guide patient selection for such therapy. Several important components of the EGFR signaling pathway have been discovered, including BRAF, PTEN, AKT and PI3K, which deserve further study as predictors of response to existing treatments, or as targets of new interventions. The unexpected detrimental outcome associated with combined EGFR and VEGF blockade is a reminder of how much there is still to learn. New combinations and novel agents will continue to shed light on how to overcome resistance to inhibitors of the EGFR pathway, and hopefully new targets will be identified. Further study of how to employ our knowledge of EGFR pathway inhibitors to improve outcomes in the adjuvant and neoadjuvant setting is also warranted. 

## Figures and Tables

**Table 1:** Clinical trials of antiEGFR monoclonal antibodies in metastatic CRC.

Study	Setting	Treatment	No. of patients	ORR (%)	mTTP (mos)	mPFS (mos)	mOS (mos)
Single Arm phase II [12]	Irinotecan-refractory	Cetuximab monotherapy	57	9	1.4	N.R.	6.4
Randomized phase II [13]	Refractory disease to 5-FU and Irinotecan	Cetuximab monotherapy vs. Cetuximab + Irinotecan	111 vs. 218	10.8* vs. 22.9	1.5* vs. 4.1	N.R.	6.9 vs. 8.6
Single Arm phase II [14]	Refractory disease to 5-FU, Irinotecan, and Oxaliplatin	Cetuximab monotherapy	346	12.4	1.4	N.R.	6.6
Single Arm phase II [15]	First-line treatment	Cetuximab + Irinotecan + 5-FU/FA	21	67	9.9	N.R.	33.0
Randomized phase III [16]	Refractory disease to 5-FU, Irinotecan and Oxaliplatin	Cetuximab monotherapy vs. BSC	287 vs. 285	8* vs. 0	N.R.	1.9* vs. 1.8	6.1* vs. 4.6
Single Arm phase II [17]	First-line treatment	Cetuximab + FOLFOX-4	43	72	N.R.	12.3	30
Randomized phase III [18]	Refractory to Oxaliplatin	Cetuximab + Irinotecan vs. Irinotecan	648 vs. 650	16.4* vs. 4.2	N.R.	4.0* vs. 2.6	10.7 vs. 10.0
Randomized phase III [19]	First-line treatment	Cetuximab + FOLFIRI vs. FOLFIRI	602 vs. 600	46.9* vs. 38.7	N.R.	8.9* vs. 8.0	N.R.
Randomized phase III [20]	Refractory disease to 5-FU, Irinotecan and Oxaliplatin	Panitumumab monotherapy versus BSC	231 vs. 232	10.0* vs. 0	N.R.	8 weeks* vs. 7.3 weeks	6.5 vs. 6.5
Randomized phase II [21]	Refractory to Irinotecan	Irinotecan + Cetuximab+ Bevacizumab vs. Cetuximab + Bevacizumab	43 vs. 40	37 vs. 20	7.3 vs. 4.9	N.R.	14.5 vs. 11.4
Single Arm phase II [22]	Refractory to Irinotecan + Bevacizumab	Cetuximab + Bevacizumab + Irinotecan	33	9	3.9	N.R.	10.6

*Statistically significant improvement.

ORR: overall response rate; mTTP: median time to progression; mPFS: median progression-free survival; mOS: median overall survival; N.R.: not reported; 5-FU: 5-fluorouracil; BSC: best supportive care; FA: folinic acid; NS: not significant.

**Table 2:** Clinical trials with retrospective subset analyses of antiEGFR efficacy in relation to KRAS mutation status.

Study	Setting	Treatment	KRAS genotype	No. of patients	ORR (%)	mPFS (mos)	mOS (mos)
Single arm studies							

Lièvre et al. [34]	Second-line treatment	Cetuximab	WT Mut	65 24	40* 0	31.4 wk* 10.1	14.3* 10.1
De Roock et al. [29]	Irinotecan refractory	Cetuximab or cetuximab + irinotecan	WT Mut	57 46	41^†^ 0	34 wk^†^ (combo) 12 (cetux) 12	44.7 wk^†^ (combo) 27 wk (cetux) 25.3–27.3
Khambata-Ford et al. [28]	Second or third-line treatment	Cetuximab	WT Mut	50 30	10 0	N.R.	N.R.
Di Fiore et al. [47]	Refractory disease	Cetuximab + chemotherapy	WT Mut	43 16	20.3 0	N.R.	N.R.
Benvenuti et al. [48]	Various lines of treatment	Cetuximab or panitumumab or cetuximab + chemotherapy	WT Mut	32 16	31 6	N.R.	N.R.

Randomized studies							

Amado et al. [24]	Refractory disease	Panitumumab + BSC vs. BSC	WT Mut vs. WT Mut	124 84 119 100	17 0 0 0	12.3 wk* 7.4 wk 7.3 wk 7.3 wk	8.1 4.9 7.6 4.4
Van Cutsem et al. [33]	First-line treatment	FOLFIRI + cetuximab vs. FOLFIRI	WT Mut vs. WT Mut	172 105 176 87	59.3 36.2 43.2 40.2	9.9* 7.6 8.7 8.1	24.9 17.5 21.0 17.7
Van Cutsem et al. [42]	First-line treatment	FOLFIRI + cetuximab vs. FOLFIRI	WT Mut vs. WT Mut	316 214 350 183	57.3* 31.3 39.7 36.1	9.9* 7.4 8.4 7.7	23.5* 16.2 20.0 16.7
Bokemeyer et al. [31]	First-line treatment	FOLFOX + cetuximab vs. FOLFOX	WT Mut vs. WT Mut	61 52 73 47	61* 33 37 49	7.7* 5.5 7.2 8.6	N.R.
Bokemeyer et al. [43]	First-line treatment	FOLFOX + cetuximab vs. FOLFOX	WT Mut vs. WT Mut	82 77 97 59	57* 34 34 53	8.3* 5.5 7.2 8.6	22.8 18.5 13.4 17.5
Karapetis et al. [35]	Refractory disease	Cetuximab + BSC vs. BSC	WT Mut vs. WT Mut	115 81 113 83	12.8 1.2 0 0	3.7* 1.8 1.9 1.8	9.5* 4.5 4.8 4.6
Siena et al. [44]	First-line treatment	FOLFOX + panitumumab vs. FOLFOX	WT = 656 Mut = 440		55 48	9.6 (wt)* 7.3 (mut) 8.0 (wt) 8.8 (mut)	N.R.
Kohne et al. [45]	First-line treatment	FOLFIRI + panitumumab	WT Mut	85 57	48 29	N.R.	N.R.
Tol et al. [49]	First-line treatment	CAPOX + bevacizumab + cetuximab vs. CAPOX + bevacizumab	WT Mut WT Mut	158 98 156 108	50.0 59.2 61.4* 45.9	10.5* 8.1 10.6 12.5	21.8 17.2 22.4 24.9
Hecht et al.[50]	First-line treatment	FOLFOX + bevacizumab + panitumumab vs. FOLFOX + bevacizumab	WT Mut WT Mut	201 135 203 125	50 47 56 44	9.8 10.4 11.5 11.0	20.7 19.3 24.5 19.3

*Statistically significant improvement

^†^Statistically significant improvement for the combination of cetuximab and irinotecan only.

ORR: overall response rate; mPFS: median progression-free survival; mOS: median overall survival; N.R.: not reported; BSC: best supportive care.
